# Editorial for Special Issue: Enzyme Immobilization and Its Applications

**DOI:** 10.3390/molecules24244619

**Published:** 2019-12-17

**Authors:** Roberto Fernandez-Lafuente

**Affiliations:** Institute of Catalysis and Petrochemistry-CSIC, Campus UAM-CSIC, C/ Marie Curie 2, Cantoblanco, 28049 Madrid, Spain; rfl@icp.csic.es

Modern chemistry demands cleaner processes, for which more efficient catalysts are required [1,2]. Enzymatic biocatalysis is among the best solutions for this demand of the society [2]. However, enzymes did not fulfil the requirements of industry, and in many instances they need to be improved. While enzyme immobilization was started just to solve the problem of enzyme recovery due to the high price of enzymes, nowadays it has become a system that may permit improvement of many enzyme features, like stability, activity, selectivity, specificity, and even purity [3,4,5,6,7]. The immobilization only will provide full advantages if analyzed in a global way, including the support and the immobilization protocol (including the active groups in the support, the immobilization conditions, and the design of a final end process) [8]. In this way, a proper enzyme immobilization remains a critical step in the industrial design of an enzymatic biocatalyst.

Enzyme immobilization is the main topic of this third Special Issue in Molecules. The previous issues gathered 45 [9] and 23 published papers [10]; in this issue, 17 new papers have been collected. The decrease in the number of papers is more related to the many Special Issues on enzyme immobilization than to a real lack of interest of the researchers in this topic [11].

The Special Issue shows a review on the characterization of the microenvironment of immobilized enzymes, a complex and critical point in understanding the immobilized enzymes [12]. Another contribution shows that the characterization of the stability of an immobilized enzyme may be better using mass of product rather than time to become inactivated [13]. This means that a proper characterization of the immobilization parameters is required [14].

In this new Special Issue, enzyme co-immobilization has become an important topic. There are a great amount of publications regarding this topic, as it presents many kinetic advantages [15,16,17,18]; however, the disadvantages tend to be ignored [6,19,20,21,22]. In this manner, horseradish peroxidase, galactose oxidase and catalase have been co-immobilized into Cu_3_(PO_4_)_2_ nanoflowers to transform hydroxymethylfurfural into 2,5-diformylfuran, avoiding the enzyme inactivation by the hydrogen peroxide produced by the oxidase and giving better yields than using individually immobilized enzymes [23]. Another paper shows the co-immobilization of a α-amylase (dextrozyme) and a protease (esperase), adding a β-galactosidase in some instances [24]. However, co-immobilization is not always an advantage. For example, the production of biodiesel was improved by using lipases from porcine pancreas and from *Thermomyces lanuginosus* that were immobilized using the crosslinked enzyme aggregate CLEA technology, but the differences in enzyme stabilities makes it inconvenient to co-immobilize both enzymes [25]. As one of the CLEAs was magnetic, they could be separated when the least stable enzyme was inactivated [25]. CLEA technology is very popular. The apparent simplicity of CLEA preparation has converted it in a widely attractive method [3,6,26].

Lipase immobilization has been a main trend in this issue. CLEA technology has been utilized to produce magnetic biocatalysts in two further contributions. In one of the examples, lipase B from *Candida antarctica* was utilized to produce magnetic CLEAs (using magnetic nanoparticles modified with 3-aminopropyltriethoxysilane) and the biocatalyst was utilized in the resolution of racemic 1-phenylethanol [27]. In the other, porcine pancreas lipase was immobilized following this technique, trying to increase the activity by tailoring the porosity of the biocatalysts using different strategies (use of polyethyleneimine or dodecyl aldehyde, co-aggregation with bovine serum albumin and/or soy protein or the addition of starch) [28].

Immobilization of lipases on hydrophobic supports is one the most used strategies to immobilize these enzymes, due to the many advantages of the process [29]. For example, lipase MAS1 was immobilized on XAD1180 resin (a hydrophobic support) and utilized to modify phosphatidylcholine with n-3 polyunsaturated fatty acids or rich ethyl esters in a solvent-free system [30]. In another research effort, the lipase from *Pseudomonas stutzeri* was immobilized on octyl silica and utilized to produce ascorbyl palmitate [31]. Lipase from *Candida rugosa* was immobilized into modified hollow mesoporous silica coated with different hydrophobic materials, with the best results being obtained using octadecyl [32]. The biocatalyst was used in the esterification of phytosterols with polyunsaturated fatty acid in a solvent-free system [32]. Lipases from *Beauveria bassiana* and *Fusarium oxysporum* were immobilized on octyl-sepharose and used to produce omega-6 and -9 acids by the hydrolysis of *Euterpe oleracea* Martius and *Mauritia flexuosa* oils, with significant hyperactivations compared to the free enzymes [33].

Immobilization of other enzymes is also treated in this Special Issue. Naranginase (presenting both α-l-rhamnosidase and β-d-glucosidase activities) was immobilized on chitosan particles activated with glutaraldehyde and utilized in the debittering of grapefruit [34]. A carbon screen-printed platform made from ceramic substrate was coated with poly(3,4-ethylenedioxythiophene)/poly(styrenesulfonate) to immobilize acetylcholinesterase and used to quantify acetylcholine [35]. In another paper, an indirect yeast surface display method was described by anchoring Im7 proteins on the surface of *Pichia pastoris*, using this system to immobilize fluorescence proteins (sfGFP and mCherry) and enzymes (human arginase I) where a CL7 fusion tag has been introduced [36]. Chemical modification (amination of the enzyme surface plus glutaraldehyde treatment) of Alcalase previously immobilized on glyoxyl agarose beads permitted significant improvement of enzyme stability, enabling the use of the enzyme at higher temperatures than the nonmodified enzyme or the free enzyme [37]. Superfolder green (sGFP) and red (RFP) fluorescent proteins have been fused with His-tags to immobilize them on graphene 3D hydrogels, with Cys-tags to immobilize them on porous matrices activated with either epoxy or disulfide groups or with Lys-tags to immobilize them on nanoparticles functionalized with carboxylic groups [38], to spatially tune the protein distribution. Finally, using silica-coated magnetic nanoparticles activated with an immobilized metal chelate or epoxide groups, single operation enrichment and immobilization of a recombinant phenylalanine ammonia-lyase from parsley fused to a poly-histidine affinity tag was accomplished, directly using the culture lysate [39].
